# Ultra-Stable Silica Nanoparticles as Nano-Plugging Additive for Shale Exploitation in Harsh Environments

**DOI:** 10.3390/nano9121683

**Published:** 2019-11-25

**Authors:** Lan Ma, Pingya Luo, Yi He, Liyun Zhang, Yi Fan, Zhenju Jiang

**Affiliations:** 1 School of Science, Xihua University, Jinzhou Road, Chengdu 610039, Sichuan, China; malan2018@mail.xhu.edu.cn; 2 State Key Lab of Oil and Gas Reservoir Geology and Exploitation, Southwest Petroleum University, 8 Xindu Avenue, Chengdu 610500, Sichuan, China; luopy@swpu.edu.cn (P.L.); 201721000242@stu.swpu.edu.cn (L.Z.); 3 College of Chemistry and Chemical Engineering, Southwest Petroleum University, 8 Xindu Avenue, Chengdu 610500, Sichuan, China; tankvan5@gmail.com; 4 Chengdu Graphene Application Institute of Industrial Technology, Leshan Road, Chengdu 610500, Sichuan, China

**Keywords:** silica, SI-ATRP, anionic polymers, brine stability, nano-plugging additive

## Abstract

Owing to the harsh downhole environments, poor dispersion of silica at high salinity and high temperature can severely restrict its application as the nano-plugging agent in shale gas exploitation. The objective of this study is to improve salt tolerance and thermal stability of silica. Herein, silica was successfully functionalized with an anionic polymer (p SPMA) by SI-ATRP (surface-initiated atom transfer radical polymerization), named SiO_2_-g-SPMA. The grafted pSPMA brushes on silica provided sufficient electrostatic repulsion and steric repulsion for stabilizing silica in a harsh environment. The modified silica (SiO_2_-g-SPMA) had excellent colloidal stability at salinities up to 5.43 M NaCl (saturated brine) and standard API brine (8 wt% NaCl + 2 wt% CaCl_2_) for 30 days at room temperature. Simultaneously, the SiO_2_-g-SPMA was stable at 170 °C for 24 h as well as stable in weakly alkali environment. Furthermore, the plugging performance of SiO_2_-g-SPMA in water-based drilling fluids for low permeate reservoir reached to 78.25% when adding a small amount of 0.5 wt% SiO_2_-g-SPMA, which effectively hindered the water invasion into formation and protected the reservoir.

## 1. Introduction

With a continuously increasing demand for energy, the depletion of conventional oil and gas urges to the exploration and development of unconventional oil and gas reserves. Shale gas is clean energy with large reserves, and the exploitation of shale gas has become a worldwide hotspot. It is known that shale formations are prone to be hydrated during shale gas drilling processes, leading to downhole complexities such as borehole collapse, tight hole, stuck pipe, etc. [1,2]. Thus, the stability of wellbore is of great importance for shale gas exploitation [3]. The effective way to minimize clay swelling is to use plugging materials, which can form a thin layer of solids to decrease water invasion into the formation to some extent. However, the existing common plugging additives including fine calcium carbonate, asphalts and walnut shell powder are all in micron-scale, the size of which was larger than nanoscale pores of shale reservoir. They cannot be plugged into the shale reservoir (low permeability reservoir), and failed to form an effective sealing layer effectively during shale gas drilling process. Hence, the traditional plugging additives cannot be used as the plugging materials in water-based drilling fluids for shale gas exploitation [4,5].

Nanoparticles, such as silica, graphene oxide, carbon nanotubes, are widely considered as the promising plugging materials for shale formations [6,7,8,9]. However, the downhole environments are very complicated at high salinity ranging from 30,000–317,000 ppm (monovalent ions) and high temperatures of 40–150 °C [10,11]. Many studies demonstrate that the existence of electrolytes made nanoparticles (oxidized MWCNTs) precipitated [12,13,14,15]. High ionic strengths have a strong tendency to make nanoparticles agglomerate due to charge screening, while elevated temperatures disrupt collision probability of particles, eventually leading nanoparticle flocculation. The nanomaterials cannot be used as the plugging agent to plug nanopores of shale formation due to aggregation.

To enhance the colloidal stability of nanoparticles in extreme conditions is crucial for its application as the nano-plugging agent for shale formations [16,17]. Previous strategies to ensure nanoparticles remain unaggregated focused on developing polymer coatings that impart steric and electrostatic stability. For instance, adding polymers in solution or grafting polymers on the nanoparticle surface via the graft to method [18,19,20]. Carlos A. Zuniga [21] found that graphene oxide (GO) was stable in standard API and Arab-D brines by grafting zwitterionic polymer on GO, however, the modified GO could only be stable below 90 °C. Mikhil Ranka [22] reported colloidal stability of silica was achieved at salt concentrations up to 120,000 ppm at 90 °C via grafting zwitterionic polymer to the silica surface. Nevertheless, few studies have been reported about the colloid stability of nanoparticles in conditions at higher ionic strength and higher temperatures above 90 °C [23,24,25,26].

**Scheme 1:**
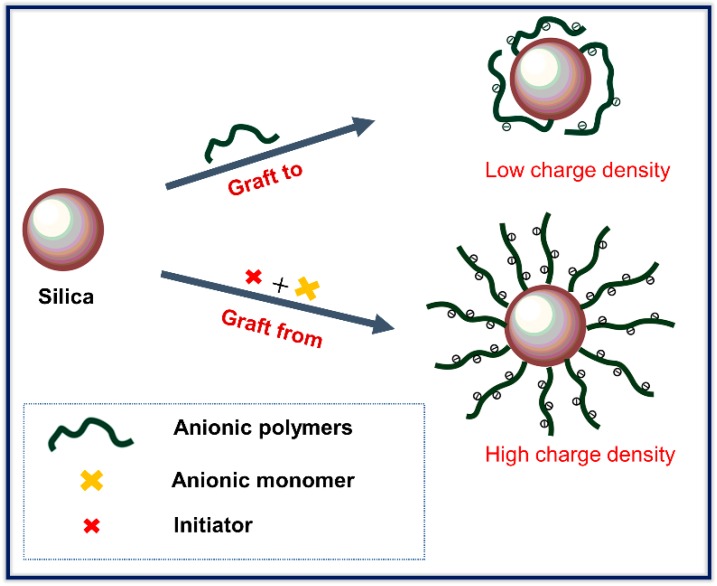
High charge density of modified silica (SiO_2_-g-SPMA) prepared via SI-ATRP method.

As mentioned above, previous studies applied the graft to approach, however, a small amount of polymer grafted on particle surface due to steric hindrance of polymers resulted in low grafting density on particle surface as well as less steric and electrostatic repulsion. This method cannot enhance the stability of silica in extreme environments. In order to improve the stability of silica in extreme environments, the graft from method, surface-initiated atom transfer radical polymerization (SI-ATRP) was devised to graft anionic polymer on silica. Well-controlled molecular weight, more polymer chains and high charge density on silica could be achieved [27,28,29] (Scheme 1) using this method. The poly (3-sulfopropyl methacrylate potassium salt) p(SPMA) with many sulfonic groups (negatively charged groups) has strong hydration capability and can resist flocculation at high temperatures. Thus, p(SPMA) can be fully stretched in aqueous solution and provide steric and electrostatic repulsion to stabilize nanoparticle suspensions [30].

In this work, the colloid stability of silica has been improved by grafting polyelectrolytes brushes on silica surface via “graft from” approach of SI-ATRP. Due to highly hydrated behavior and massive negative charges of anionic polyelectrolytes (pSPMA), the electrostatic repulsion and steric hindrance of polymer chains were constructed, which endowed the silica nanoparticles with high stability in harsh environments. The modified silica NPs displayed long-term stability in saturated brine of 5.43 mol NaCl as well as the high temperature of 170 °C. Simultaneously, a laboratory evaluation method was proposed to investigate the plugging performance of the nano-plugging agent, and the modified silica NPs exhibited excellent plugging performance for low permeating formations with 78.25% plugging efficiency. It is shown that the modified silica is a promising nano-plugging agent in water-based drilling fluids for low permeating formation.

## 2. Experimental Section

### 2.1. Materials

Fumed silica (SiO_2_, fumed, purity >99.8%, diameter 30–40 nm, Aladdin), Copper(I)bromide (Cu(I)Br, 99%), 2-bromoisobutyryl bromide (2-BIBB, 97%), *N*,*N*,*N*′,*N*′,*N*″-pentamethyldiethylenetriamin (PMDETA, 99%) and 3-sulfopropylmethacrylate (SPMA, 98%) in the form of a potassium salt were purchased from Aladdin (Chengdu, China). Hydrogen chloride solution (HCl), sodium chloride (AR, 99.5%), calcium chloride (AR, 99.5%), toluene, methanol, dimethyl sulfoxide (DMSO), tetrahydrofuran (THF), and amino-propyltriethoxysilane (APTES) were purchased from the Kelong company (Chengdu, China). Tetrahydrofuran (THF) was freshly distilled and collected immediately prior to use. Copper(I)bromide (Cu(I)Br, 99%) was deoxidized by acetic acid and washed with methanol before use. Other chemicals were used as received without further purification.

### 2.2. Preparation of p(SPMA) Brushes-Grafted MWCNTs

#### 2.2.1. Immobilization of Initiator onto Silica (SiO_2_-APTES-Br)

Firstly, silica nanoparticles were activated by the HCl solution, namely SiO_2_-OH. Then the SiO_2_-OH were modified with 3-aminopropyl triethoxysilane (APTES) and named SiO_2_-APTES. The above processes are described specifically in Supplementary Materials 2.2. Afterward, the SiO_2_-APTES (800 mg) was dispersed in dry tetrahydrofuran (THF, 100 mL). Triethylamine (TEA, 0.05 mol) was added in the above solution. After cooling to 0 °C, 2-bromoisobutyryl bromide (BIBB, 0.08 mol) was added drop by drop stirring at room temperature for 12 h. After the reaction, the 2-bromoisobuty-functionalized silica nanoparticles were washed with absolute ethanol and dried in a vacuum oven overnight at 60 °C. The initiator-functionalized silica nanoparticles named SiO_2_-APTES-Br. The number of grafted initiators was estimated at 0.074 mmol per 100 mg SiO_2_-APTES-Br according to TGA results (Supplementary Materials 2.2).

#### 2.2.2. Grafting pSPMA Brushes on Silica (SiO_2_-g-SPMA)

The initiator (SiO_2_-APTES-Br, 100 mg, 0.074 mmol of initiating sites) were dispersed in the mixed solvents of 9 mL distilled water and 6 mL DMSO and sonicated for 30 min. The solution was degassed and charged with Ar (g). Then 3-sulfopropylmethacrylate (SPMA, 0.011 mol) and *N*,*N*,*N*′,*N*′,*N*″-pentamethyl diethylenetriamine (PMDETA, 0.15 mmol) were added in above solution, fresh Cu(I)Br (0.074 mmol) was added. During the process of polymerization, the temperature was maintained at 60 °C in an oil bath. After polymerization, the mixture was purified in a dialysis tube (molecular weight cutoff at 10 kDa) with distilled water for 10 days and was freeze-dried. In this work, SiO_2_-g-SPMA was synthesized with the molar ratios of [SiO_2_-APTES-Br]:[SPMA]:[PMDETA]:[Cu(I) Br] = 1:150:2:1.

### 2.3. Characterization

The modified silica composites were characterized by infrared spectroscopy instrument (WQF-520, Beijing Rayleigh Analytical Instrument Co., Ltd, China) and X-ray photoelectron spectroscopy (XPS, Escalab 250Xi, Thermo Fisher Scientific, Waltham, MA, USA). Morphologies of modified silica and the filter cakes were measured using the transmission electron microscopy (TEM) (JEOL 2010F, Tokyo, Japan) and the environmental scanning electron microscope (ESEM) (FEI Quanta 450, USA). The organic contents of modified silica were determined by thermogravimetric analysis by a TGA (TGA i1000, Instrument Specialists Inc, Twin Lakes, WI, USA) at a heating rate of 10 °C/min from 40 °C to 800 °C.

### 2.4. Colloid Stability in Electrolytes

A series of dispersions (1 mg/mL) of SiO_2_-g-SPMA nanocomposite in different NaCl concentration solutions (0, 4, 10, 20, 30 wt%), including API brines (8 wt% NaCl + 2 wt% CaCl_2_) were prepared. The visual inspection was recorded by digital images. Particle sizes of SiO_2_-g-SPMA in the prepared solutions were determined by dynamic light scattering (DLS) on a Zetasizer Nano-ZS particle analyzer (Zeta PALS 190 Plus, Brookhaven, NY, USA). All particle sizes data were the average of five measurements. The zeta-potential values of SiO_2_-g-SPMA dispersed in water (at the concentration of 1 mg/mL) at different pH values were determined by a Zetasizer Nano-ZS particle analyzer (Zeta PALS 190 Plus, Brookhaven, NY, USA). The instrument measures the electrophoretic light scattering of a 35 mW solid-state laser beam at a 660 nm wavelength, and zeta potential were the means of 3–5 measurements.

### 2.5. Plugging Test

To evaluate the micro-nano plugging performance of SiO_2_-g-SPMA nanocomposites, the low permeating filter cakes (permeability reach to 10^−4^ mD) as the low permeating core samples were prepared firstly. Detailed information is presented in the Supporting Information.

The plugging tests of SiO_2_-g-SPMA were conducted on the above prepared low-permeating filter cake (LPFC) with micropores and nanopores. Firstly, a series of aqueous dispersions with 0.1, 0.2, 0.3, 0.4, and 0.5 wt% SiO_2_-g-SPMA were prepared. Then the different concentration SiO_2_-g-SPMA dispersions were respectively added in the HTHP filter, allowing the SiO_2_-g-SPMA dispersion to flow through above low-permeating filter cake (LPFC) at 105 °C under a pressure of 3.5 MPa. Afterward, the volumes of filtrate at each 5 min interval were recorded, sustaining for 30 min. When the test was finished, the fresh filter cake was carefully removed from the HTHP filter and digital images were taken immediately. The thickness of the filter cake was also confirmed by an electronic caliper. After adding SiO_2_-g-SPMA as the nano-plugging agent, the permeability (K′) of filter cake was also calculated according to Darcy’s Law (referred to above calculation method in Supplementary Materials (Equation S1).

(1) $$K_{r}=\frac{K_{0}-K^{\prime }}{K_{0}}\times 100\%$$

where *K*_r_ represented the reduction rate of permeability; *K*_0_ was the mean permeability of low-permeating filter cake (LPFC); *K*′ represented the permeability of filter cake after adding nano-plugging agent.

## 3. Results and Discussion

### 3.1. Characterization of Modified Silica

**Scheme 2:**
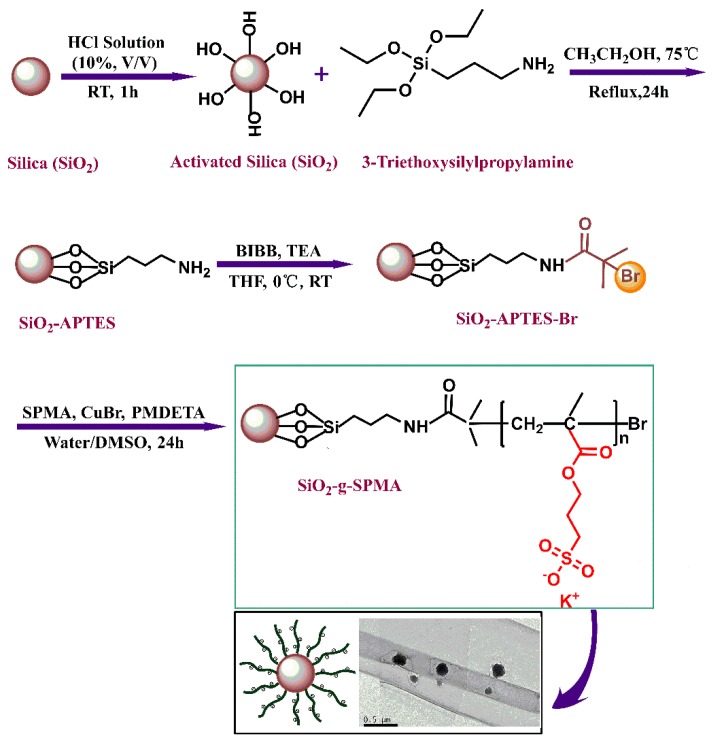
Preparation of SiO_2_-g-SPMA by surface-initiated atom transfer radical polymerization (SI-ATRP) method.

The strategy devised for modifying silica to improve dispersibility at high ionic concentration and high temperature is schematically illustrated in Scheme 2. Silica was firstly activated by the HCl solution and coated with APTES. Then, initiators (BIBB) were anchored on the silica surface via an amide reaction. At last, polymers (pSPMA) were grafted on silica surface via SI-ATRP. The TEM image in Scheme 2 represented the polymers modified silica. The green halo around the particles was the structure of grafted polymers on silica after drying in air. During TEM test, SiO_2_-g-SPMA dispersions were dried in the air, resulting in silica particles gathering, therefore, larger particles than the size of original unmodified silica were detected. The aggregation also existed during the SiO_2_-g-SPMA preparation due to the van der Waals interparticle attraction, so some large size particles were observed.

**Figure 1:**
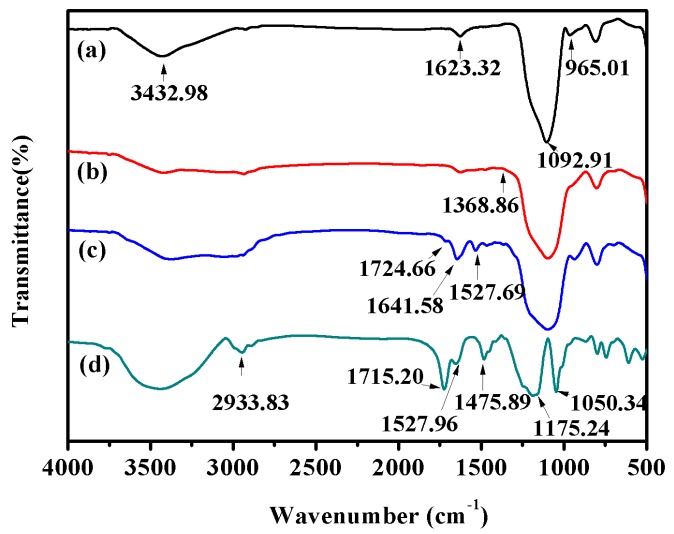
FTIR spectra of modified silica, (**a**) SiO_2_-OH, (**b**) SiO_2_-APTES, (**c**)SiO_2_-APTES-Br, and (**d**) SiO_2_-g-SPMA.

**Figure 2:**
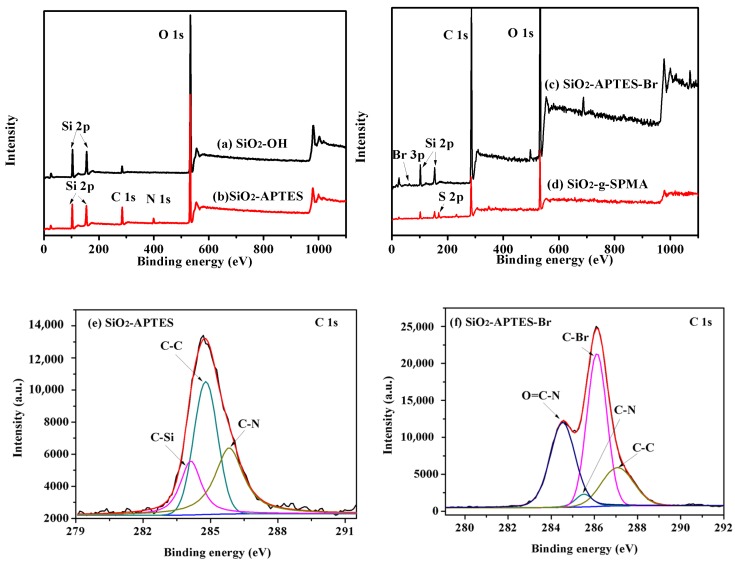
(**a**–**d**) The broad scans spectra of SiO_2_-OH, SiO_2_-APTES, SiO_2_-APTES-Br and SiO_2_-g-SPMA; (**e**) C 1s spectrum for SiO_2_-APTES; and (**f**) C 1s spectrum for SiO_2_-APTES-Br.

After modification, SiO_2_-OH, SiO_2_-APTES, SiO_2_-APTES-Br, and SiO_2_-g-SPMA were obtained and their physiochemical properties and microstructures were characterized by FTIR, XPS, TGA, SEM, TEM, and ESEM. Figure 1a shows the FTIR spectrum of activated silica (SiO_2_–OH), peaks at 800 and 1100 cm^−1^ for Si−O, 965 cm^−1^ for Si−OH, and the broad range of 3200 to 3700 cm^−1^ for OH are observed in SiO_2_–OH. After surface modification with APTES, the new peak at 1385 cm^−1^ assigned to the –CH_2_, the stretching of C–H at 2933 cm^−1^ assigned for alkanes, and the bending of N–H at 694 cm^−1^ were detected (Figure 1b). Simultaneously, the Si−OH peak at 965 cm^−1^ was significantly decreased. The above results indicated that APTES was successfully grafted onto the silica through chemical bonding. In Figure 1c, a new peak at 1724 cm^−1^ was attributed to the C=O group from amide, indicating that the initiator 2-bromoisobutyryl bromide (BIBB) was successfully immobilized on the surface of silica. In the spectrum of SiO_2_-g-SPMA (Figure 2d), two new bands at 1175 and 1050 cm^−1^ were attributed to the S=O stretching vibration of sulfonate groups from the SPMA monomer [31,32], confirming the successful grafting of pSPMA brushes onto the surface of SiO_2_ nanoparticles via ATRP method.

The general XPS spectra of SiO_2_-OH and SiO_2_-APTES (Figure 2a,b) showed that the new peaks at 399 eV belonged to N 1s appeared in SiO_2_-APTES. In Figure 2c, a new peak at 68–69 eV in general XPS spectra of SiO_2_-APTES-Br was attributed to Br 3d. Furthermore, the high-resolution C1s spectrum of SiO_2_-APTES (Figure 2e) was fitted into three peaks at 284.1, 284.8, and 285.8 eV, representing the binding energy of C–Si, C–C and C–N, respectively and inferring that APTES were successfully coated onto the surface of silica. As shown in Figure 2f, C1s spectra of the initiator SiO_2_-APTES-Br was fitted with four components with binding energies of 284.5, 285.5, 286.1, and 287.0 eV, which correspond to C–C, C–N, C–Br, and N–C=O, respectively [33]. The peak of N–C=O (287.0 eV) indicated that the successful amide reaction between 2-bromoisobutyryl bromide (BIBB) and SiO_2_-APTES occurred. Sulfur (S 2p, 168 eV) and potassium (K 2p, 293 eV) peaks observed in general spectra of SiO_2_-g-SPMA (Figure 2d) and 4.01% K atom in SiO_2_-g-SPMA (Table S1) further proved that p(SPMA) was successfully grafted on silica via SI-ATRP.

**Figure 3:**
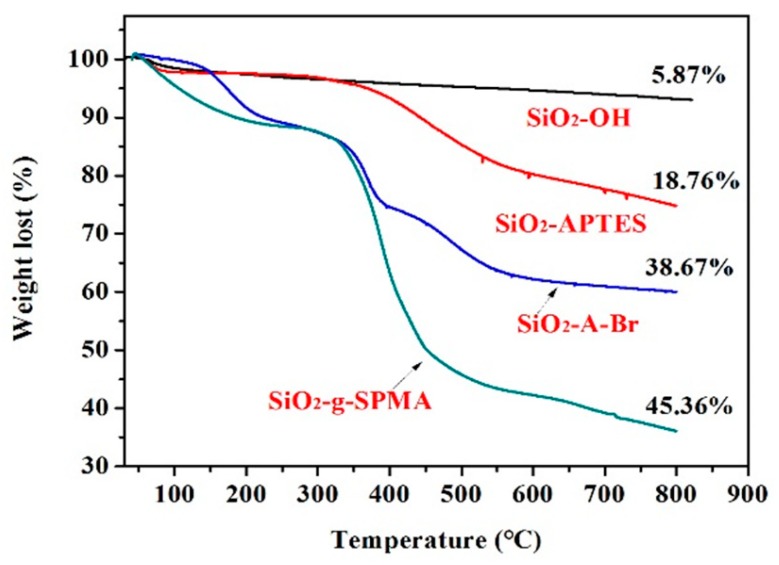
TGA curves of various modified silica.

The thermal stability and organic contents of modified silica (SiO_2_-OH, SiO_2_-APTES, SiO_2_-APTES-Br, and SiO_2_-g-SPMA) were analyzed by TGA (Figure 3). Compared to the weight loss of SiO_2_-OH, an obvious weight loss of 18.76% and 38.67% for respective SiO_2_-APTES and SiO_2_-APTES-Br between 100 and 800 °C. Therefore, a high density of initiator sites on silica was confirmed. After the initiator grafted on silica, the increased weight loss of 10.95% for SiO_2_-APTES-Br compared to SiO_2_-APTES between 150 and 380 °C was obtained. The change in weight loss between SiO_2_-APTES and SiO_2_-APTES-Br was due to the BIBB grafting. Based on the TGA results of SiO_2_-APTES and SiO_2_-APTES-Br, the grafting density of the initiator group was estimated as ca. 0.074 mmol per 100 milligrams of SiO_2_-APTES-Br. TGA analysis of SiO_2_-g-SPMA suggested that the number of pSPMA brushes grafted on silica surface was about 6.69 wt%.

**Figure 4:**
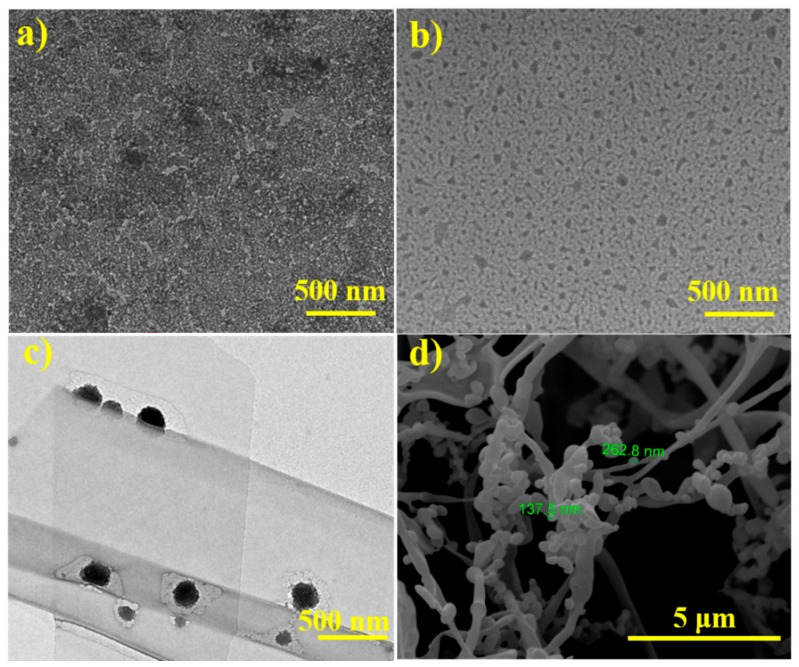
(**a**) TEM images of silica, (**b**) TEM images of SiO_2_-g-SPMA composite, (**c**) TEM image of SiO_2_-g-SPMA composite in distilled water, and (**d**) ESEM images of SiO_2_-g-SPMA composite.

The morphology of original silica and SiO_2_-g-SPMA was observed by TEM and ESEM. Figure 4a,b represented the unmodified silica and SiO_2_-g-SPMA, respectively. The unmodified silica presented a spherical structure (Figure 4a) [34]. After modification, network structures of polymers-wrapped on silica were observed (Figure 4b). The TEM images (Figure 4a,b) showed the particle size of unmodified silica was smaller than the SiO_2_-g-SPMA, and aggregation of the unmodified silica was observed. The morphologies of the unmodified silica and SiO_2_-g-SPMA demonstrated that SiO_2_-g-SPMA was successfully prepared. In aqueous solution, the polymer layers of SiO_2_-g-SPMA were somewhat more shallow and transparent color than spherical silica particles [35,36], and the nanoparticles became a little larger and exhibited core–shell-like structure (Figure 4c), suggesting the good dispersion of SiO_2_-g-SPMA in aqueous solution. Simultaneously, the spherical silica grafted with a lot of polymer chains structures were observed in ESEM image of SiO_2_-g-SPMA (Figure 4d), and diameter of SiO_2_-g-SPMA particles were estimated at the range of 100 to 300 nm. Compared to unmodified silica, the larger size of SiO_2_-g-SPMA was attributed to the grafting polymer shells. Therefore, p(SPMA) polymers successfully grafted on silica surface by SI-ATRP method were further confirmed.

### 3.2. Colloidal Stability

**Figure 5:**
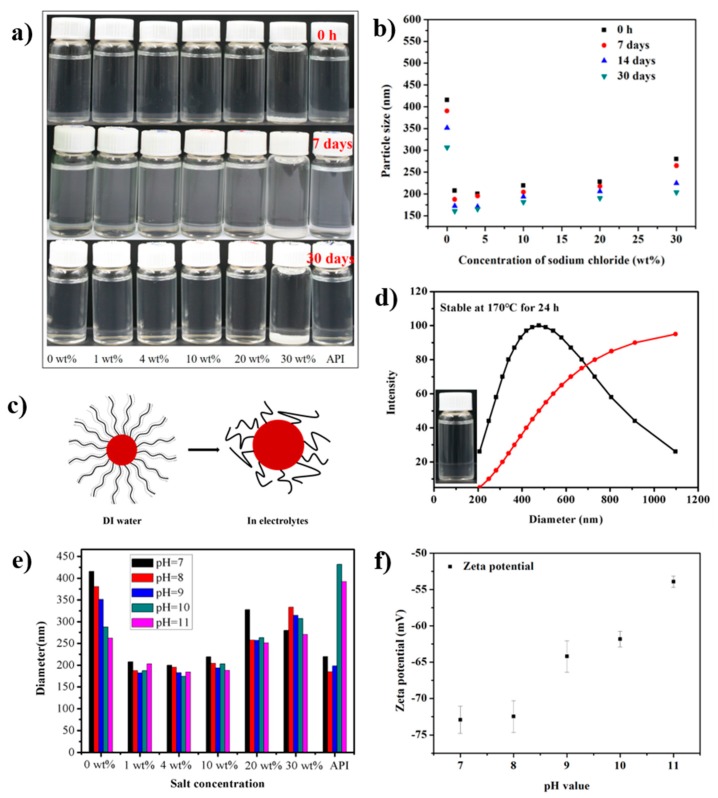
(**a**) Digital images of SiO_2_-g-SPMA dispersions in various concentration electrolytes for 0, 7 and 30 days, respectively (all above dispersions were 1 mg/mL). (**b**) Diameter of SiO_2_-g-SPMA in different concentration electrolytes with different settling time (0, 7, 14, 30 days). (**c**) Molecular conformation changes of SiO_2_-g-SPMA in distilled water and in electrolytes. (**d**) Diameter of SiO_2_-g-SPMA stabilized in saturated brine at 170 °C for 24 h. (**e**) Diameter of SiO_2_-g-SPMA in electrolytes at different pH values. (**f**) Zeta potential values of SiO_2_-g-SPMA aqueous dispersion at different pH values.

To evaluate the colloid stability of modified silica in electrolytes, SiO_2_-g-SPMA were dispersed in different concentration of NaCl brines and API brine. And the concentration of SiO_2_-g-SPMA was 1.0 mg/mL. Remarkably, after modification, SiO_2_-g-SPMA could be well dispersed in distilled water and certain high concentrated brines (30 wt% NaCl), including API brine (Figure 5a). For comparison, the dispersion of unmodified silica (1.0 mg/mL) in 2.0 wt% NaCl solution for 12 h almost completely settled (Figure S1a), and the mean diameter of unmodified silica in 2.0 wt% NaCl solution reached to 4.099 μm (Figure S1b). Furthermore, SiO_2_-g-SPMA could be stable in a salt solution (even at a salinity of 30 wt% NaCl and API brine) for 30 days (Figure 5a), suggesting long-term stability of SiO_2_-g-SPMA in concentrated brines.

The average hydrodynamic diameter of SiO_2_-g-SPMA in concentrated electrolytes was further confirmed by dynamic light scattering (DLS). The average hydrodynamic diameter of SiO_2_-g-SPMA particles was about 415 ± 10 nm in aqueous solution at initial preparation, which was larger than SiO_2_-g-SPMA particles in a salt solution (Figure 5b). The hydrophilic polymer chains grafted on silica were highly hydration and fully stretched without salts, thus, larger particle sizes were measured. However, the electrostatic repulsion interactions and interchain repulsion of polymer chains were weakened after adding salts due to negatively charges screened by salts, resulting in the polymers chains of modified silica curled and particle size decreased. The polymer conformation changes of SiO_2_-g-SPMA particles in distilled water and in electrolytes were depicted in Figure 5c.

Moreover, the average hydrodynamic diameters of SiO_2_-g-SPMA dispersed in salt solutions varied with ionic strength (Figure 5b), which was attributed to the conformational changes of the brush polyelectrolytes affected by the ionic strength [37]. When the salt concentration was below 4.0 wt%, the hydrodynamic diameter of polyelectrolytes was decreased with increasing ionic strength. For high salt concentrations (above 10.0 wt% NaCl), the polymer brushes’ behavior was affected by the electrostatic interactions of chains and the excluded volume of the polymer backbone [38,39]. The positive excluded volume led to an increased thickness of brushes, thus the hydrodynamic diameters of particles were increased with increasing ionic strength owning to the excluded volume effect in high salt concentrations (above 10.0 wt% NaCl) (Figure 5b) [40,41]. Nevertheless, the average hydrodynamic diameters of SiO_2_-g-SPMA dispersed in API brines and 30 wt% NaCl solution was kept at 219 ± 2 and 280 ± 8 nm, respectively. The results demonstrated that anionic polymer-modified silica possessed excellent colloidal stability in concentrated brine.

The long-term stability of SiO_2_-g-SPMA in concentrated brine was also important for its application in the oil and gas exploitation industry. The SiO_2_-g-SPMA in saturated brines and API brines kept stability with no appreciable aggregates for 30 days (Figure 5a). The average diameters of SiO_2_-g-SPMA in a salt solution with different settling time were also measured. The average diameter of SiO_2_-g-SPMA decreased with the extension of settling time, indicating the chains of polyelectrolytes were compressed over time (Figure 5b). The average diameter of SiO_2_-g-SPMA dispersed in 30 wt% NaCl and API brine over 30 days were kept at 203 ± 4 and 299 ± 2 nm, respectively, indicating the modified silica had the long-term stability in concentrated electrolytes.

The water-based drilling fluids were utilized in weakly alkali environment and at high temperature (>80 °C), therefore, the stability of SiO_2_-g-SPMA as a function of high temperature and alkalinity (saturated brine) was required to be investigated. The experiments of colloidal stability at high temperature were conducted in the hydrothermal reactor and stabilized for 24 h. Then, the colloidal stability of the suspension was observed after the temperature decreased to room temperature. SiO_2_-g-SPMA dispersed in saturated brines at 170 °C for 24 h had no obvious settlement and the particle sizes were about 423 ± 8 nm (Figure 5d), demonstrating the sulfonic groups derived from grafted p(SPMA) on the surface of silica were much resistant to high temperature, which was nearly twice as higher as in previous reported (at 90 °C) [42]. When the temperature increased to 180 °C, the colloid stability of SiO_2_-g-SPMA in saturated brines was destroyed. The possible reason was the grafting polymer chains (p(SPMA)) started to degrade at high temperature (>180 °C), the charge density of silica was decreased, and the electrostatic repulsion and steric repulsion effect were weakened, leading to instability of dispersions.

The diameter of SiO_2_-g-SPMA in various concentrate electrolytes was below 450 nm and had slight changes at the pH range of 7–11 (Figure 5e), indicating SiO_2_-g-SPMA was well dispersed in electrolytes as well as weak alkalinity. In the colloidal system, zeta potential (ζ-potential) was an important index, which was related to the stability of the colloidal dispersion and indicated the degree of repulsion between adjacent, similarly charged particles in a suspension [43]. The zeta potential of SiO_2_-g-SPMA was about—(73 ± 7) mV at the pH of 7 in water, the zeta potential values kept at—(54 ± 5) mV at the pH of 11 in water (Figure 5f). The high negative charge density of SiO_2_-g-SPMA was attributed to the SI-ATRP (graft from) method, resulting in multi-site grafting on the silica surface. It was suggested SiO_2_-g-SPMA had a lot of negative charges due to massive sulfate groups (−O−SO_3_^−^) in poly (3-sulfopropyl methacrylate potassium salt) brushes. The massive sulfate groups were prone to be hydration, resulting in polymers expanded and providing steric repulsion for particle stability. With the increasing pH values, the zeta potential values decreased gradually, suggesting the surface charge was screened by the increasing ionic strength. Nevertheless, the zeta potential values kept at—(54 ± 5) mV at the pH of 11, inferring that stability of SiO_2_-g-SPMA dispersions was mainly ascribed to the steric repulsion effect. The above results suggested colloid stability of SiO_2_-g-SPMA was extraordinarily stable in a weak alkali environment (pH = 7–11).

### 3.3. Plugging Performance of Modified Silica for Low Permeability Reservoir

**Figure 6:**
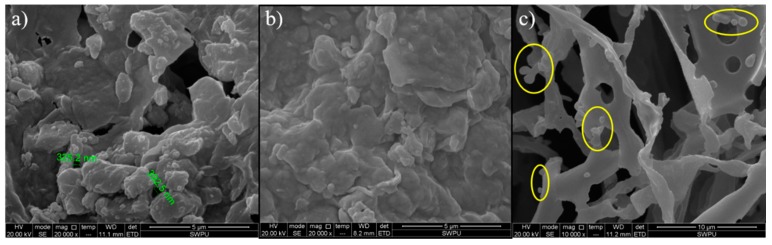
(**a**) ESEM image of prepared low permeability filter cake without modified silica, (**b**) ESEM image of filter cake with 0.5 wt% SiO_2_-g-SPMA composites, (**c**) ESEM images of filtrate with SiO_2_-g-SPMA.

Before the plugging test for low permeability reservoir, low permeability filter cakes (LPFC) were prepared to simulate the low permeability shale formation. The specific preparation process of low permeability filter cakes (LPFC) was depicted in Supplementary Materials 2.5.1. According to Darcy’s law, the average permeability (K_0_) was about 4.69 × 10^−4^ mD, indicating the low permeability of LPFC was consistent with the permeability of shale formation (Table 1). The pores and hole sizes of prepared LPFC approximately ranged from a hundred nanometers to a few micrometers investigated by ESEM (Figure 6a), which were attributed to nano- or micro-scale pores and holes. The above results indicated that our prepared LPFC was able to be simulated as the low permeability formation for plugging performance evaluation of SiO_2_-g-SPMA.

To study the plugging performance of SiO_2_-g-SPMA, different concentration (0.1, 0.2, 0.3, 0.4, 0.5 wt%) of SiO_2_-g-SPMA aqueous dispersions were prepared. In Table 2, the permeability of filter cakes (K′) decreased with adding the SiO_2_-g-SPMA dispersions, in addition, the decrease of permeability was more obvious with increasing of SiO_2_-g-SPMA amount. When the amount of SiO_2_-g-SPMA increased to 0.5 wt%, the permeability of filter cake decreased by 78.25%, whereas Chenevert and Sensoy group reported that the dosage of unmodified nano-silica for plugging the nanopores of shale formation is up to 29 wt% [44]. Compared to the previous study, SiO_2_-g-SPMA exhibited outstanding plugging performance for LPFC. The SiO_2_-g-SPMA was stable without aggregation during the HTHP filtration test. Therefore, the modified silica was convinced to be served as a high-performance nano-plugging agent in the water-based drilling fluid, retarding the filtrate into low permeable formations and protecting the reservoirs from being damaged effectively.

Afterwards, the morphologies of LPFC without and with 0.5 wt% SiO_2_-g-SPMA were observed by ESEM images (Figure 6a,b). Compared to the ESEM images of LPFC, a more compact filter cake has been formed and the pore sizes of filter cake significantly decreased after adding modified SiO_2_-g-SPMA. According to micromorphology analysis, the SiO_2_-g-SPMA could be served as the effective plugging materials for low permeating filter cakes. However, some SiO_2_-g-SPMA particles were detected in filtrate (Figure 6c), indicating that some smaller modified silica could pass through the large holes without trapped [45], also suggesting that the SiO_2_-g-SPMA dispersions were stable at 105 °C under a pressure of 3.5 MPa.

### 3.4. The Plugging Mechanism

**Figure 7:**
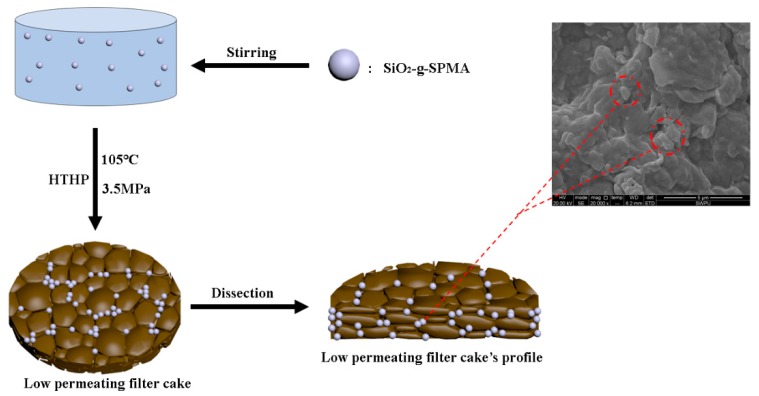
The plugging test of SiO_2_-g-SPMA for low permeating formation (shale formations).

The plugging test of SiO_2_-g-SPMA for low permeable formation has been depicted in Figure 7. During the HTHP filtration test, the SiO_2_-g-SPMA particles as the plugging materials blocked the micro or nano-pore throats and cracks. For smaller size pores and holes, the modified silica might be embedded in the pores or laid in the cracks under high pressure to form tight mud cake. For larger pores, the sizes of these pores were larger than the size of modified silica; in that case, the modified silica SiO_2_-g-SPMA could bridge and stack at the entrance of pores [46]. As the increasing amount of SiO_2_-g-SPMA, the tubes stacked more tightly, the more compact filter cakes were formed, substantially permeability of filter cakes was decreased. It was noteworthy that the excellent plugging performance for LPFC was attributed to good dispersion of SiO_2_-g-SPMA nanoparticle in a downhole environment, thus the particle size was in accordance with the pore size of LPFC. Therefore, the colloidal stability of SiO_2_-g-SPMA in drilling fluids (extremely environment) was a vital process for its application as the nano-plugging agent for low permeability formations (shale formation). Herein, the results demonstrated that SiO_2_-g-SPMA was well stable in concentrated brine (about 5.43 M NaCl) at high temperature, which exhibited excellent plugging performance as the nano-plugging materials for low permeability formations.

## 4. Conclusions

In this work, the colloidal stabilization of nano-silica has been greatly improved at extreme salinity and high-temperature conditions by grafting anionic polymer (pSPMA) via SI-ATRP method. After modification, silica was grafted with a lot of polymer chains, which provided silica with efficient steric repulsion for stabilizing silica in extreme environments. The modified silica (SiO_2_-g-SPMA) was able to be dispersed automatically in saturated brines (about 5.43 M NaCl) and API brine at room temperature for at least 30 days, as well as stable in saturated brines at 170 °C for 24 h. Additionally, the excellent plugging performance of SiO_2_-g-SPMA for low permeating reservoir (shale formation) was about 78.25% even at a low concentration of 0.5 wt% SiO_2_-g-SPMA. Overall, highly stable silica in the extreme environments (high electrolytes, high temperature, alkali) were obtained in this work, promoting the practical application as the nano-plugging agent for shale oil and gas exploitation.

## Figures and Tables

**Table 1:** The average-permeability of prepared low permeability filter cakes (LPFC).

Test	FL_initial_/mL	FL_final_/mL	Q/(cm^3^/s)	d/mm	K/mD
1	1.6	6.0	2.07 × 10^−3^	2.05	4.76 × 10^−4^
2	1.5	5.7	2.16 × 10^−3^	1.95	4.51 × 10^−4^
3	1.7	5.9	2.24 × 10^−3^	2.0	4.79 × 10^−4^
Average-permeability (K_0_) = 4.69 × 10^−4^ mD					

**Table 2:** Effect of various amount of SiO_2_-g-SPMA on plugging performance for low permeating filter cakes.

Content/wt%	FL_initial_/mL	FL_final_/mL	Q/(cm^3^/s)	d/mm	K′/mD	Kr (%)
0.1%	1.2	5.4	1.61 × 10^−3^	2.05	3.54 × 10^−4^	24.73
0.2%	1.2	5.0	1.44 × 10^−3^	1.95	2.86 × 10^−4^	39.02
0.3%	1.7	5.9	1.08 × 10^−3^	2.00	2.31 × 10^−4^	51.81
0.4%	1.4	5.6	5.57 × 10^−4^	2.05	1.22 × 10^−4^	73.99
0.5%	1.3	4.0	5.0 × 10^−4^	1.90	1.02 × 10^−4^	78.25

## Supplementary Materials

The following are available online at https://www.mdpi.com/2079-4991/9/12/1683/s1, Figure S1: (a) Digital images of silica in distilled water and 2.0 wt% NaCl solution for 12 h, (b) Diameter of silica in 2.0 wt% NaCl solution after 12 h. Table S1: Atom composition of modified silica (SiO_2_-OH, SiO_2_-APTES, SiO_2_-APTES-Br and SiO_2_-g-SPMA).
