# Supplementary material for: Ultra-Stable Silica Nanoparticles as Nano-Plugging Additive for Shale Exploitation in Harsh Environments
Source: Nanomaterials (Basel). 2019 Nov 25;9(12):1683. doi: 10.3390/nano9121683 (PMC6955846; doi:10.3390/nano9121683)
Supplement: Supplementary file 1 [file nanomaterials-09-01683-s001.pdf]

Ultra-Stable Silica Nanoparticles as Nano-Plugging Additive for Shale Exploitation in Harsh Environments

Lan Ma [1,2], Pingya Luo [2,3], Yi He [2,3,*], Liyun Zhang [2,3], Yi Fan [3,4] and Zhenju Jiang [1,*]

1 School of Science, Xihua University, Jinzhou Road, Chengdu 610039, Sichuan, China; malan2018@mail.xhu.edu.cn
2 State Key Lab of Oil and Gas Reservoir Geology and Exploitation, Southwest Petroleum University, 8 Xindu Avenue, Chengdu 610500, Sichuan, China; luopy@swpu.edu.cn (P.L.); 201721000242@stu.swpu.edu.cn (L.Z.)
3 College of Chemistry and Chemical Engineering, Southwest Petroleum University, 8 Xindu Avenue, Chengdu 610500, Sichuan, China; tankvan5@gmail.com
4 Chengdu Graphene Application Institute of Industrial Technology, Leshan Road, Chengdu 610500, Sichuan, China
* Correspondence: chemheyi@swpu.edu.cn (Y.H.); jiangzhenju@sina.com (Z.J.)

2. Experimental section

2.2. Preparation of p(SPMA) brushes-grafted MWCNTs

Activation of silanol groups (SiO_2-OH) Firstly, silica nanoparticle (6 g) were added in a 500 mL three neck beaker, then dispersed in 100 mL of HCl (10%, v/v). The reaction was kept for 1h at room temperature. The activated silica NPs was obtained by washing with water three times and evaporation of the solvent in vacuum drying oven. The yield of activated silica NPs (SiO_2-OH) was 83%.

Modification Silica with APTES (SiO_2-APTES) Activated silica NPs (SiO_2-OH) (1.6 g) were dispersed in 100 mL alcohol (95%), then 5 mL ammonia (28 wt%) and APTES (4.0 g) were added in above dispersion. the mixture was refluxed at 75 °C for 24 h. The whole process was under nitrogen protection. Then, the mixture was washed with alcohol at least 3 times to remove unreacted APTES. The products were dried in a vacuum oven at 60 °C for 24 h and named SiO_2-APTES.

Calculation process of grafted initiator amounts After initiator grafted on silica, the increased weight loss of 10.95% for SiO_2-APTES-Br compared to SiO_2-APTES between 150 and 380 °C was obtained. And the change weight loss between SiO_2-APTES and SiO_2-APTES -Br was due to the BIBB grafting. Based on the TGA results of SiO_2-APTES and SiO_2-APTES-Br, the initiating group density of SiO_2-APTES-Br was estimated as ca. 0.074 mmol per 100 milligrams of SiO_2-APTES-Br.

2.5. Plugging test

2.5.1. Preparation of low permeating filter cakes (LPFC)

At first, sodium carbonate (5 wt% based on bentonite) was dispersed in 10 wt% bentonite base fluids. Fluid loss-control additives (5 wt% SMC + 5 wt% SMP-1) were added and stirred for 20 min. Subsequently, 100 g barite ($m_{API\ barite}$: $m_{nano\text{-}barite}$ = 7:3) was added and stirred for 1 h, then, the bentonite base fluids were placed overnight and spared.

Secondly, the above prepared bentonite base fluids were poured into a high temperature and high-pressure filter press equipment (HTHP filter, GGS42-2, Qing Dao) and filtration tests were carried out at 105 °C under a pressure of 3.5 MPa. The volumes of initial filtrate and final filtrate (30 min) were recorded during the measurement. After the test finished, the loose filter cake was carefully removed without damage, and the low permeating filter cake was obtained. To comfirm that the permeability of filter cake reached to 10^{-4} mD, the permeability of filter cake was measured as the followings: A volume of distilled water was added in the above formed filter cake, allowing

the distilled water to flow through the filter cake at 105 °C under a pressure of 3.5 MPa. Afterwards, the volumes of filtrate at each 5 min interval were recorded, sustaining for 30 min. When the test was finished, the fresh filter cake was carefully removed from the HTHP filter and digital images were taken immediately. The thickness of filter cake was also measured by an electronic caliper. The permeability (K) of a fresh filter cake was calculated according to Darcy's Law:

$$K = \frac{Q\mu L}{A\Delta P} \quad \text{(S1)}$$

Where Q was the filtration rate (cm^3/s), the volume of collected filtrate versus time to obtain a straight line with a slope value, which corresponded to the Q value; μ is the viscosity of filtrate at 25 °C (1 cP); L was the thickness of filter cake (mm); ΔP was the total pressure drop (3.5 MPa); A was the cross-sectional area to fluid flow (23.8 cm^2), which can be measured using the liquid flowing through already formed filter cake. The mean permeability (K_0) value of low-permeating filter cakes (LPFC) was obtained by three parallel experiments tests.

3. Results and discussion

The surface atom composition of SiO_2-OH, SiO_2-APTES, SiO_2-APTES-Br, and SiO_2-g-SPMA were obtained by XPS. The atom composition of modified silica was shown in Table S1. After modification with APTES, the 3.40% N atom was detected in the surface of SiO_2-APTES. The 3.23% Br atom was detected in SiO_2-APTES-Br due to the grafting of 2-bromoisobutyryl bromide (BIBB) on SiO_2-APTES. After polymerization, only 0.28% Br atom was detected in SiO_2-g-SPMA, whereas 4.01% K atom and 3.97% S atom detected in SiO_2-g-SPMA, that were the results of p(SPMA) successfully grafted on silica via SI-ATRP.

Table S1 Atom composition of modified silica (SiO_2-OH, SiO_2-APTES, SiO_2-APTES-Br and SiO_2-g-SPMA).

	Element	Atom composition (%)
SiO_2-OH	C 1s	3.98
	O 1s	84.06
	Si 2p	11.96
SiO_2-APTES	C 1s	10.4
	O 1s	74.45
	Si 2p	11.74
	N 1s	3.40
SiO_2-APTES-Br	C 1s	30.34
	O 1s	55.20
	Si 2p	7.85
	N 1s	3.38
	Br 3d	3.23
SiO_2-g-SPMA	C 1s	33.75
	O 1s	50.03
	Si 2p	4.15
	N 1s	3.81
	K 2p	4.01
	S 2p	3.97
	Br 3d	0.28

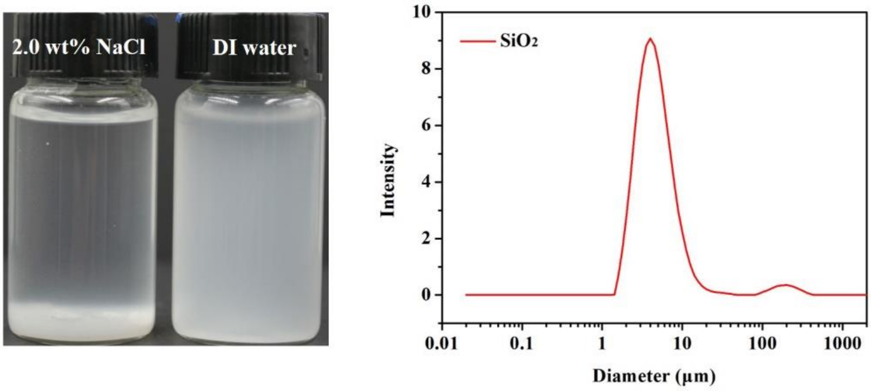

(a) **(b)**

Figure S1 (a) Digital images of silica in distilled water and 2.0 wt% NaCl solution for 12 h, (b) Diameter of silica in 2.0 wt% NaCl solution after 12 h. (All above dispersions were 1 mg/mL).

To evaluate the colloid stability of unmodified silica in electrolytes, the dispersion of unmodified silica (1.0 mg/mL) in distilled water and 2.0 wt% NaCl solution were prepared. Figure S1 showed the digital images of silica in distilled water and 2.0 wt% NaCl solution for 12 h. The silica was highly stable in distilled water for 12 h. However, the silica dispersed in 2.0 wt% NaCl almost completely settled after 12 h (Figure S1a). And the mean diameter of unmodified silica in 2.0 wt% NaCl solution after 12 h reached to 4.099 μm (Figure S1b).
